# A Newly Discovered Tendon Between the Genioglossus Muscle and Epiglottic Cartilage Identified by Histological Observation of the Pre-Epiglottic Space

**DOI:** 10.1007/s00455-022-10469-7

**Published:** 2022-06-09

**Authors:** Kei Kitamura, Tae Watanabe, Masahito Yamamoto, Noboru Ishikawa, Norio Kasahara, Shinichi Abe, Hitoshi Yamamoto

**Affiliations:** 1 Department of Histology and Developmental Biology, Tokyo Dental College, 2-9-18 Kandamisaki-cho, Chiyoda-ku, Tokyo 101-0061 Japan; 2 Department of Anatomy, Tokyo Dental College, 2-9-18 Kandamisaki-cho, Chiyoda-ku, Tokyo 101-0061 Japan; 3 Department of Forensic Odontology and Anthropology, Tokyo Dental College, 2-9-18 Kandamisaki-cho, Chiyoda-ku, Tokyo 101-0061 Japan

**Keywords:** Pre-epiglottic space, Epiglottic cartilage, Genioglossus muscle, Anatomy

## Abstract

Epiglottic retroversion is difficult to explain anatomically. One reason is inadequate structural identification of the ligaments in the submucosal tissue anterior to the epiglottis (pre-epiglottic space, PES). Although studies have shown that tongue root movement plays a role in epiglottic retroversion, few morphological reports have investigated the attachment of the lingual muscles to the epiglottis. This study reconstructed the fiber structure of the PES by comprehensively analyzing fiber alignment in the PES focusing on the hyoepiglottic ligament, which runs between the lingual muscles and the epiglottis. Gross and microscopic observations of the submucosal structures from the tongue to the larynx of 20 cadavers (10 men, 10 women; mean age 79 years) were performed. A tendon continuing from the posterior part of the genioglossus muscle and attaching to the center of the epiglottic cartilage was identified in the midline area of the epiglottis. We named this tendon the glossoepiglottic tendon. In contrast, the hyoepiglottic ligament is found between the hyoid bone and the epiglottis and is attached from the lateral margin of the epiglottic cartilage to its base. Furthermore, the glossoepiglottic tendon consists of a high-density fiber bundle that is thicker than the hyoepiglottic ligament. These results show that the conventional hyoepiglottic ligament has a two-layer structure consisting of an upper fiber bundle connected to the genioglossus muscle and a lower fiber bundle connected to the hyoid bone. Sustained contraction of the posterior part of the genioglossus muscle therefore places the epiglottis under persistent traction, suggesting that its relaxation may cause epiglottic retroversion.

## Introduction

The epiglottis is a process that points superiodorsally from the root of the tongue at the boundary between the pharynx and the larynx. It shifts backward during swallowing to cover the laryngeal aperture in the movement known as the swallowing reflex [1], preventing the food bolus from being carried into the airway. The swallowing reflex is an involuntary movement, and if the reflex is mistimed, the food bolus may be carried into the airway, causing dysphagia (aspiration) [2, 3]. Conventionally, dysphagia was mostly regarded as a sequela of amyotrophic lateral sclerosis and other motor neuron diseases, but age-related structural changes around the epiglottis are also attracting attention as another cause [4–6]. Sarcopenia, a condition characterized by a generalized decrease in muscle mass, is considered to be one of the major conditions facilitating aspiration, since the mass and function of the lingual muscles associated with swallowing are severely diminished [7–10]. The functional and physiological relationships between the lingual muscles and the epiglottis have thus been well reported. However, there have been few morphological reports concerning the attachment of the lingual muscles to the epiglottis.

**Fig. 1:**
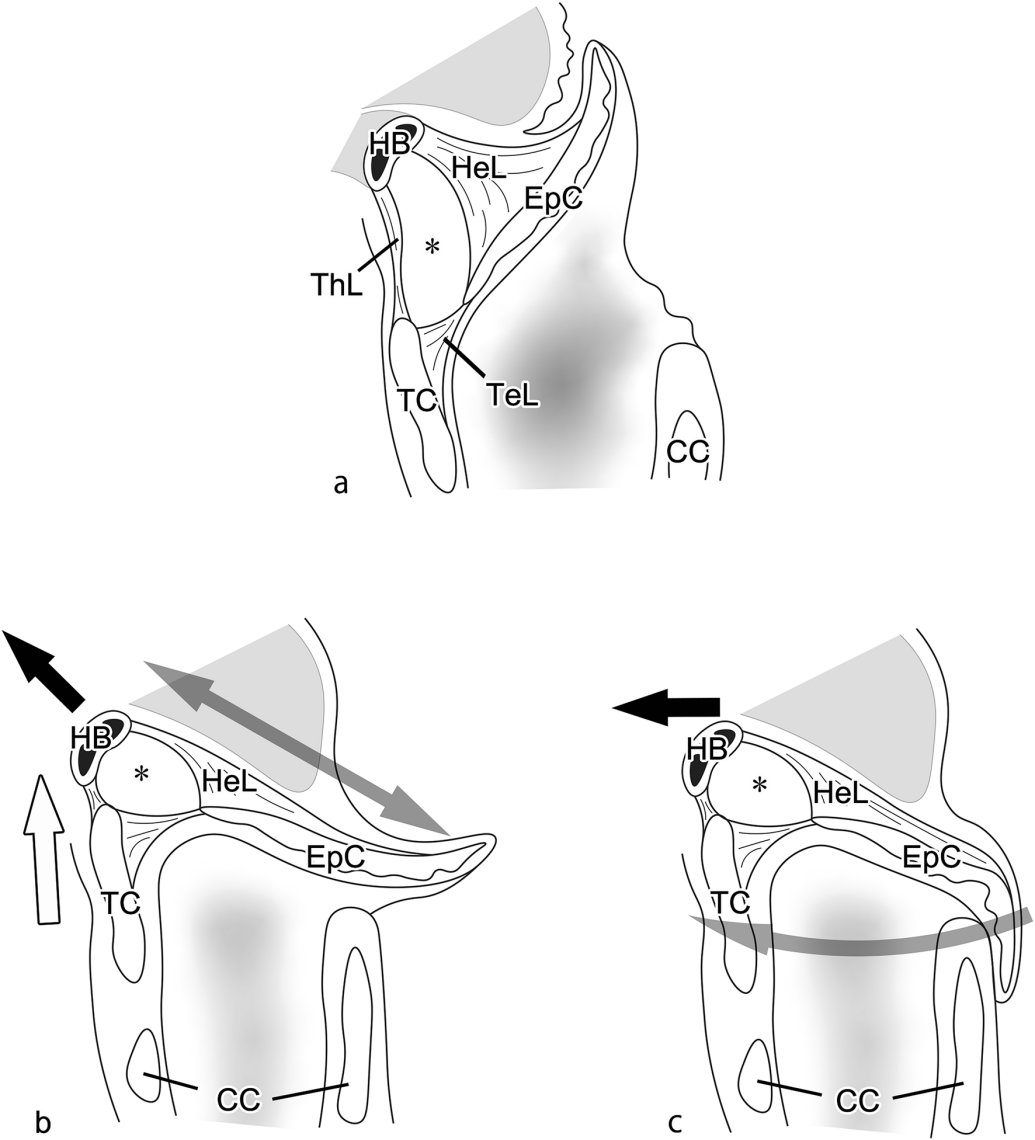
Schematic drawing of a median sagittal section of the larynx and epiglottic inversion. The submucosal area anterior to the epiglottis (the pre-epiglottic space, PES) is formed by the hyoepiglottic ligament (HeL), which connects the epiglottic cartilage (EpC) to the hyoid bone (HB); the thyrohyoid ligament (ThL), which connects the thyroid cartilage (TC) to the HB; the thyroepiglottic ligament (TeL), which connects the EpC to the TC; and the pre-epiglottic adipose tissue (*), which is found within this area (**a)**. The inversion of the epiglottis during swallowing starts with the first epiglottic movement, in which the HeL at the midline is extended (gray arrow) by the forward movement of the HB (black arrow) and the upward movement of the larynx (white arrow), and the epiglottis becomes horizontal (**b**). Then, in the second epiglottic movement, as a result of the forward movement of the HB (black arrow), the lateral HeL places the tip of the epiglottis under traction (gray arrow), so that its top third folds over (**c**). CC: cricoid cartilage

The skeleton of the epiglottis is made up of cartilage with high elastic fiber content, and this epiglottic cartilage is connected to the tissues around it by a number of ligaments. At the front of the epiglottis, these include (1) the hyoepiglottic ligament, which connects the epiglottis to the hyoid bone; (2) the thyrohyoid ligament, which connects the hyoid bone to the thyroid cartilage; and (3) the thyroepiglottic ligament, which connects the epiglottis to the thyroid cartilage. The space surrounding these ligaments is known as the pre-epiglottic space (PES) [11–13] (Fig. 1a). The PES is known to be an extremely important structure that enables epiglottic retroversion, with the hyoepiglottic ligament in particular performing complex actions during the short reflex. The normal swallowing reflex mechanism comprises the first epiglottic movement at the start of retroversion, in which the epiglottis reaches the horizontal position (Fig. 1b), and the second epiglottic movement, in which the top third of the epiglottis folds downward to cover the laryngeal aperture completely (Fig. 1c). In the first epiglottic movement, the hyoepiglottic ligament extends to support the retroversion of the epiglottis, but in the second epiglottic movement, it draws the tip of the epiglottis forward together with the hyoid bone [14–17]. To explain these actions of the hyoepiglottic ligament, Van Daele et al*.* showed that the hyoepiglottic ligament is attached not only to the hyoid bone but also to the fascia of the root of the tongue, suggesting that the hyoepiglottic ligament may consist of more highly active tissue. Reidenbach further named the fiber bundle running toward the tongue root the “hyoepiglottic membrane” and showed that the hyoepiglottic ligament and hyoepiglottic membrane are fiber bundles running anteriorly from the lingual surface of the epiglottic cartilage [18]. Recent studies have reported that the fibrous components of the hyoepiglottic ligament and hyoepiglottic membrane are decreased by smoking and aging, and the degradation of these fibers has been implicated in obstructive sleep apnea and dysphagia [19, 20]. However, the hyoepiglottic membrane is a thin-fiber bundle belonging to the hyoepiglottic ligament, and it is unclear whether it is a structure capable of controlling the movements of the epiglottis.

In this study, a comprehensive visualization of the fibrous structures present in the PES was conducted, with a focus on the hyoepiglottic ligament, with the objectives of identifying new fiber bundles and clarifying the functional and physiological mechanisms of epiglottic movements from the morphological perspective.

## Materials and Methods

This study was conducted in compliance with the provisions of the 1995 Declaration of Helsinki (revised in Edinburgh in 2000). Twenty donated cadavers (10 men and 10 women) of mean age 79 years (range 72–88 years) were studied. These cadavers were donated to Tokyo Dental College for use in human dissection research and teaching, and their use in this study was approved by the university’s ethics committee (approval no. 891). The donated cadavers were fixed by arterial perfusion with 10% v/v formalin solution and preserved for more than 3 months with 70% v/v ethanol solution. Tissue blocks were prepared containing the tongue, epiglottis, thyroid cartilage, arytenoid cartilage, and upper cricoid cartilage. All samples were decalcified by incubation in 0.5 mol/l EDTA solution (pH 7.5; decalcifying solution B, Wako, Tokyo, Japan) for 30 days at room temperature. Paraffin embedding was carried out by the normal method, with cut planes comprising 10 samples in sagittal section, 5 in horizontal section, and 4 in coronal section (with all sections including both men and women). From each of the prepared paraffin blocks, four 5–10-μm-thick semi-continuous sections at approximately 0.3-mm intervals were prepared. Two of these prepared sections were stained with hematoxylin and eosin (H–E) stain and the other two with elastica-Masson stain (a variation of Masson–Goldner staining). The tissue slices were observed and photographed with a universal photomicroscope (UPM Axiophot 2, Carl Zeiss Microscopy, Jena, Germany), but ultra-low-magnification photographs (less than 1 × at the objective lens) were photographed with a high-grade flat scanner (Epson GTX970; Seiko Epson Corporation, Nagano, Japan) equipped with translucent lighting.

Scanning electron microscopy (SEM) specimens were obtained from a single cadaver. The tongue root and epiglottis were harvested from the cadaver *en bloc*, and the epithelium was selectively removed under stereomicroscopy. The sample was immersed for 1 h in 10% sodium hypochlorite solution and rinsed in 30% hydrogen peroxide. It was then dried to the critical point of t-butyl alcohol, and evaporation coating was conducted with a carbon coater (VC-100S, Vacuum Device, Mito, Japan). Finally, the morphology of the fiber courses was observed by SEM (SU6600, Hitachi High-Tech, Tokyo, Japan).

## Results

### Gross Anatomical Observations of the Tongue and Epiglottis

**Fig. 2:**
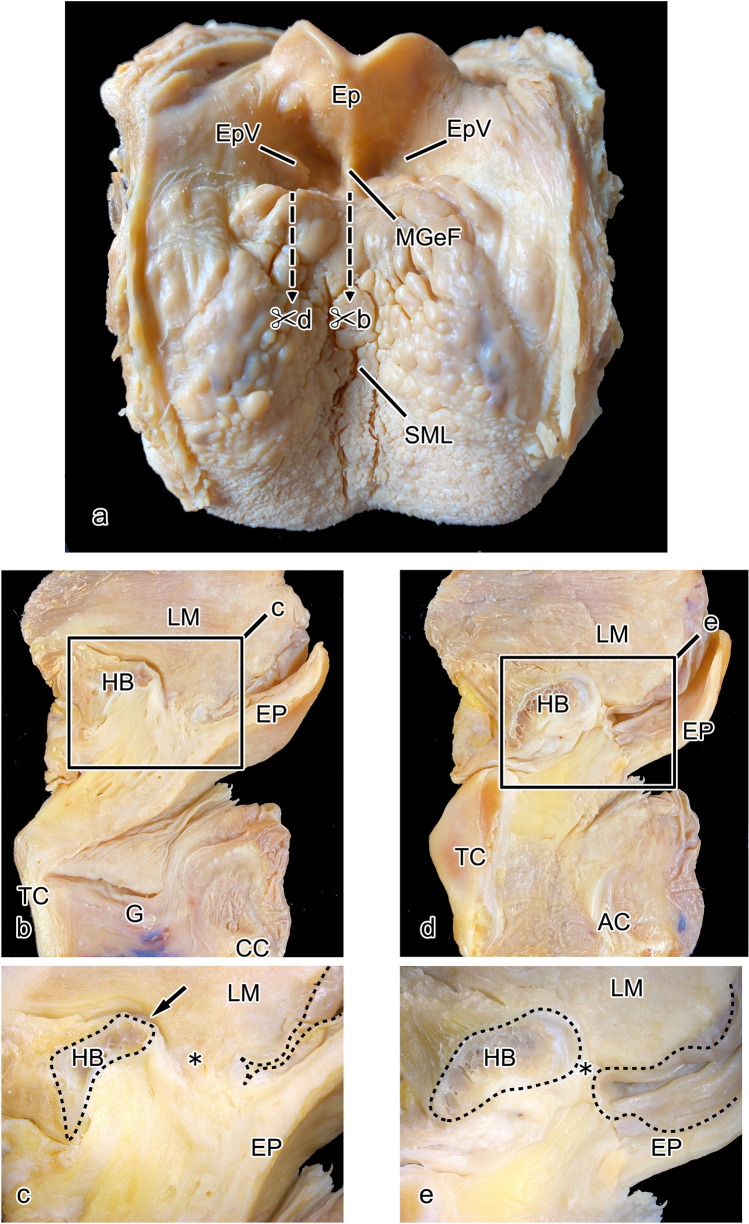
Gross dissections of the tongue and epiglottis. From above, the median glossoepiglottic fold (MGeF) is evident anterior to the EP, with the epiglottic valleculae (EpVs) on either side (**a**). In sagittal section of the MGeF, the hyoid bone (HB) can be seen to be inferior and anterior to the lingual muscles (LM) and the EP to be inferior and posterior to them, and at the next level down, the thyroid cartilage (TC) is observed anterior to the glottis, whereas the cricoid cartilage (CC) is posterior to it (**b**). In the magnified image of the area within the box, thick submucosal tissue is evident between the epithelium and the HB (**c**, asterisk), and a slit extends posteriorly from the upper part of the HB (**c** arrow). In the sagittal section of the EpV, there is no change to the positions of the LMs, HB, EP, and TC, but the glottis has disappeared, and the arytenoid cartilage (AC) is newly apparent (**d**). In the magnified image of the area within the box, the area between the epithelium and the HB has changed to thin submucosal tissue (**e**, asterisk), and there is no slit above the HB. SML: sulcus medianus linguae

Gross dissection was first performed to observe the structure of the subsurface mucosal tissue in the PES. When the tongue and epiglottis were viewed from above, bilaterally symmetrical epiglottic valleculae were evident anterior to the epiglottis. Between the valleculae, the median glossoepiglottic fold ran as a continuation of the sulcus medianus linguae on the dorsum of the tongue (Fig. 2a). In median sagittal section (Fig. 2a, cutting line b), the lingual muscles were evident above the epiglottis, the hyoid bone anterior to the epiglottis, and the glottis inferior to the epiglottis, with the thyroid cartilage anterior to the glottis and the cricoid cartilage posterior to the glottis (Fig. 2b). In the magnified image of the area within the box, a slit-shaped structure was formed above the hyoid bone (Fig. 2b, c, arrow), and thick submucosal tissue was present between the hyoid bone and the epithelium (Fig. 2c, asterisk). In the lateral sagittal section (Fig. 2a, cutting line d), muscle tissue ran between the thyroid cartilage and the arytenoid cartilage, and the glottis was not apparent (Fig. 2d). In the magnified image of the area within the box in this cross-section, there was no evident slit-like structure in the upper part of the hyoid bone, and the submucosal tissue between the hyoid bone and the epithelium was thinner (Fig. 2e, asterisk).

## Histological Analysis of the Tongue and Epiglottis Observed in Sagittal Sections

### Median Sagittal Section

**Fig. 3:**
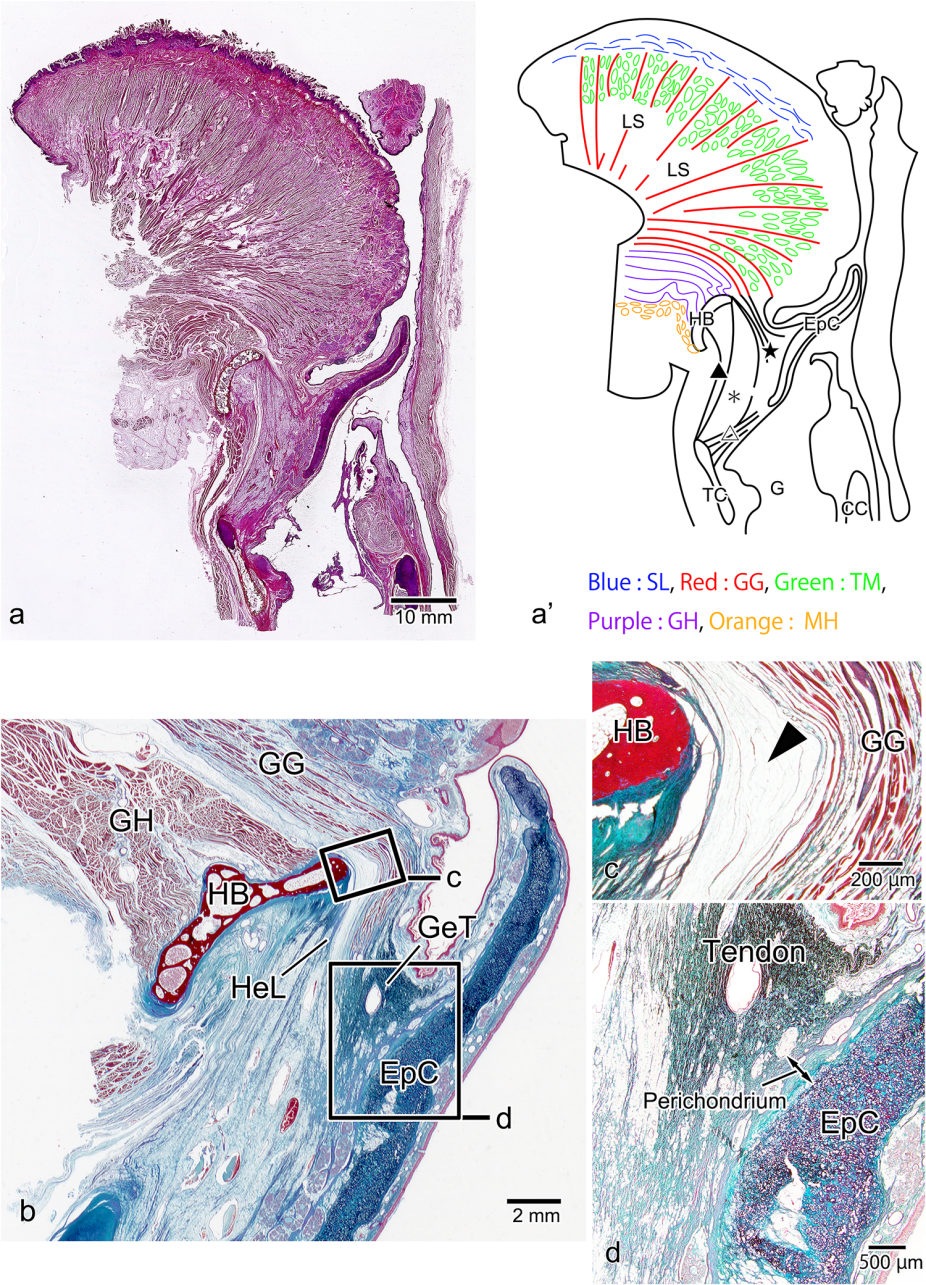
Histological observations of the tongue and epiglottis in median sagittal section. In the surface layer of the tongue, the superior longitudinal (Blue: SL) lingual muscle runs from the tip of the tongue to the dorsal tongue, and within the tongue, the genioglossus (Red: GG) muscle runs in a fan shape from the deep part of the tongue toward its surface (**a**, a’). In the magnified image of the tongue root, the posterior portion of the GG muscle passes through the upper part of the geniohyoid (Purple: GH) muscle and is transformed into the glossoepiglottic tendon (GeT) behind the hyoid bone (HB). The hyoepiglottic ligament (HeL) is observed from the superior edge of the dorsal surface of the HB to the base of the epiglottic cartilage (EpC) (**b**). A slit with sparse connective tissue is formed between the GG muscle and the HB (**c**). The GeT has high elastic fiber content and is broadly attached to the perichondrium of the EpC (**d**). *TM* transverse muscle of the tongue (Green), *MH* mylohyoid muscle (Orange), *LS* lingual septum, *TC* thyroid cartilage, *G* glottis, *CC*: cricoid cartilage, ★: hyoepiglottic ligament, filled triangle: thyrohyoid ligament, open triangle: thyroepiglottic ligament, *: pre-epiglottic adipose tissue

In the median sagittal section of the epiglottis, the superior longitudinal lingual muscle was observed from the tip of the tongue to the subsurface mucosal layer of the dorsum of the tongue. In the deep layer of the superior longitudinal lingual muscle, the genioglossus muscle, which passed between the transverse muscle of the tongue and the lingual septum, ran in a fan shape along the entire surface of the tongue (Fig. 3a, a’). In the magnified image of the tongue root, the posterior-most portion of the genioglossus muscle passed through the upper part of the geniohyoid muscle to reach the dorsal surface of the hyoid bone and from the upper tip of the hyoid bone the hyoepiglottic ligament ran toward the base of the epiglottic cartilage (Fig. 3b). A slit containing almost no connective tissue was present between this genioglossus muscle and the hyoid bone (Fig. 3c, arrowhead). The genioglossus muscle was also transformed into a tendon with a high elastic fiber content, which had a broad attachment to the perichondrium of the epiglottic cartilage. This tendon corresponds to the hyoepiglottic membrane of the midline, but since it is a fiber bundle independent of the hyoepiglottic ligament, we named it the glossoepiglottic tendon (Fig. 3d).

### Sagittal Sect. 3-mm Lateral to the Midline (Mid-Lateral)

**Fig. 4:**
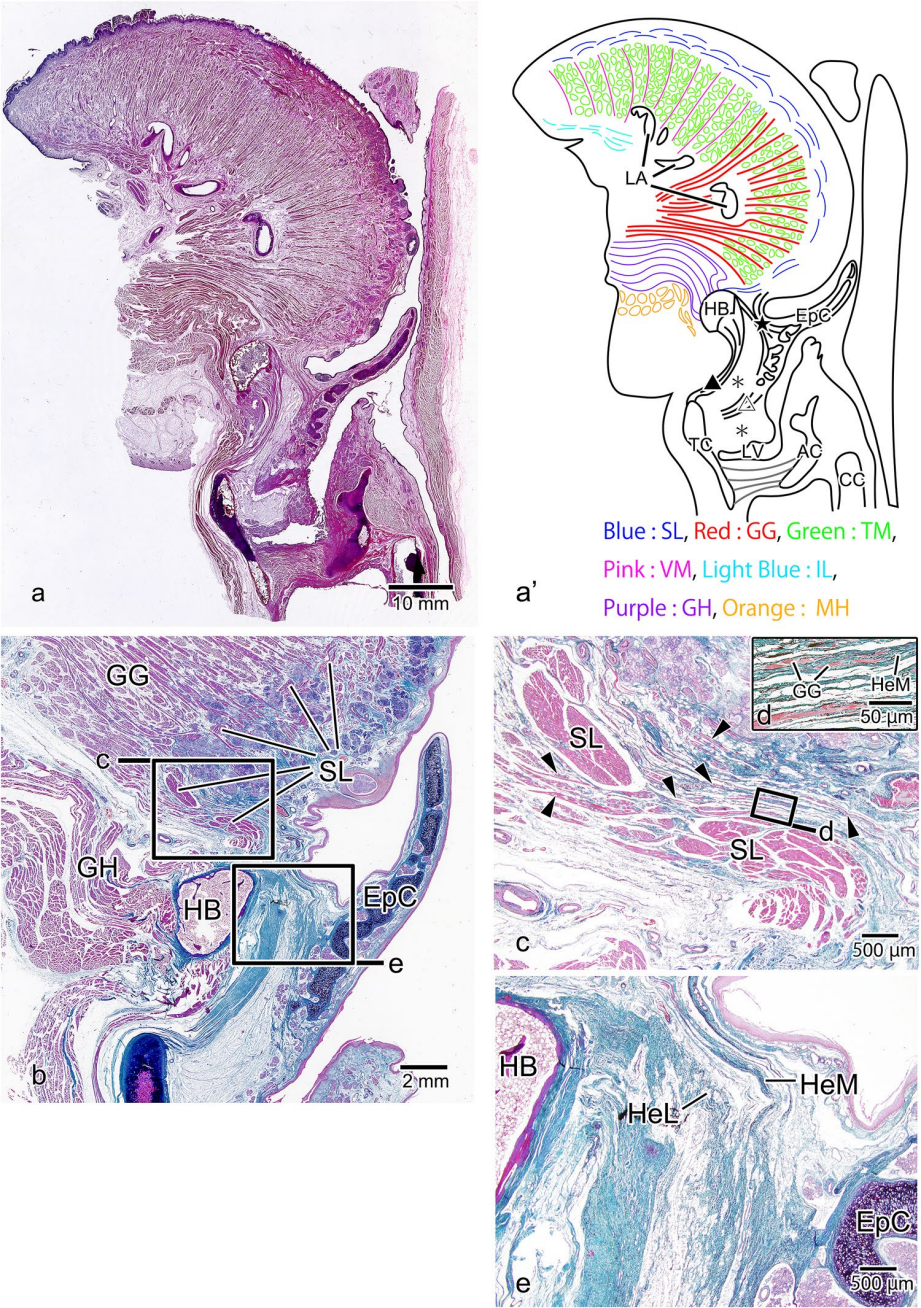
Sagittal Sect. 3-mm lateral to the midline. In the surface layer of the tongue, the superior longitudinal lingual (SL: Blue) muscle runs from the tip of the tongue and attaches to the hyoid bone (HB). Within the tongue, the genioglossus (GG: Red) muscle is evident in the posterior tongue, but in the anterior part of the tongue, it is progressively replaced by the vertical muscle (VM: Pink) and the transverse muscle (TM: Green) of the tongue and the inferior longitudinal (IL: Light Blue) lingual muscle (**a**, a’). In the magnified image of the tongue root, because the SL lingual muscle that originates from the hyoid bone separates the GG muscle from the epiglottic cartilage (EpC), the glossoepiglottic tendon (GeT) is not evident (**b**). However, some of the muscle fibers of the GG muscle go between the muscle bundles as they run toward the larynx (**c**, arrowhead). The GG muscle fibers that have penetrated in this way are transformed into the hyoepiglottic membrane (HeM) (**d**). The HeM merges with the hyoepiglottic ligament (HeL) and attaches to the EpC. *LA* lingual artery, *LV* larynx ventricle, *TC* thyroid cartilage, *CC* cricoid cartilage, *AC* arytenoid cartilage, *GH* geniohyoid muscle　(Purple), *MH*: mylohyoid muscle (Orange), ★: hyoepiglottic ligament, filled triangle: thyrohyoid ligament; open triangle: thyroepiglottic ligament; *: pre-epiglottic adipose tissue

In a sagittal Sect. 3-mm lateral to the median sagittal section, the superior longitudinal lingual muscle was evident beneath the epithelium from the tip of the tongue to the tongue root, finally terminating at the superior edge of the hyoid bone. In its deep part, the genioglossus muscle was only evident in a limited area from the center of the dorsum of the tongue to the posterior tongue, running in a serpentine fashion to avoid the lingual artery and the transverse muscle of the tongue. The vertical muscle and the inferior longitudinal muscle of the tongue were present in the anterior part of the genioglossus muscle (Fig. 4a, a’). In the magnified image of the tongue root, no obvious glossoepiglottic tendon forming a muscle–tendon junction with the genioglossus muscle was apparent, unlike in Fig. 2, because of the superior longitudinal lingual muscle running between the tongue root and the epiglottis (Fig. 4b). However, the muscle fibers in the posterior-most part of the genioglossus muscle went between the superior longitudinal lingual muscle bundles (Fig. 4c, arrowheads) and were transformed into the collagenous hyoepiglottic membrane (Fig. 4d). The hyoepiglottic membrane merged with the hyoepiglottic ligament between the hyoid bone and the epiglottic cartilage, running toward the base of the epiglottic cartilage (Fig. 4e).

#### Sagittal Sect. 6-mm Lateral to the Midline

**Fig. 5:**
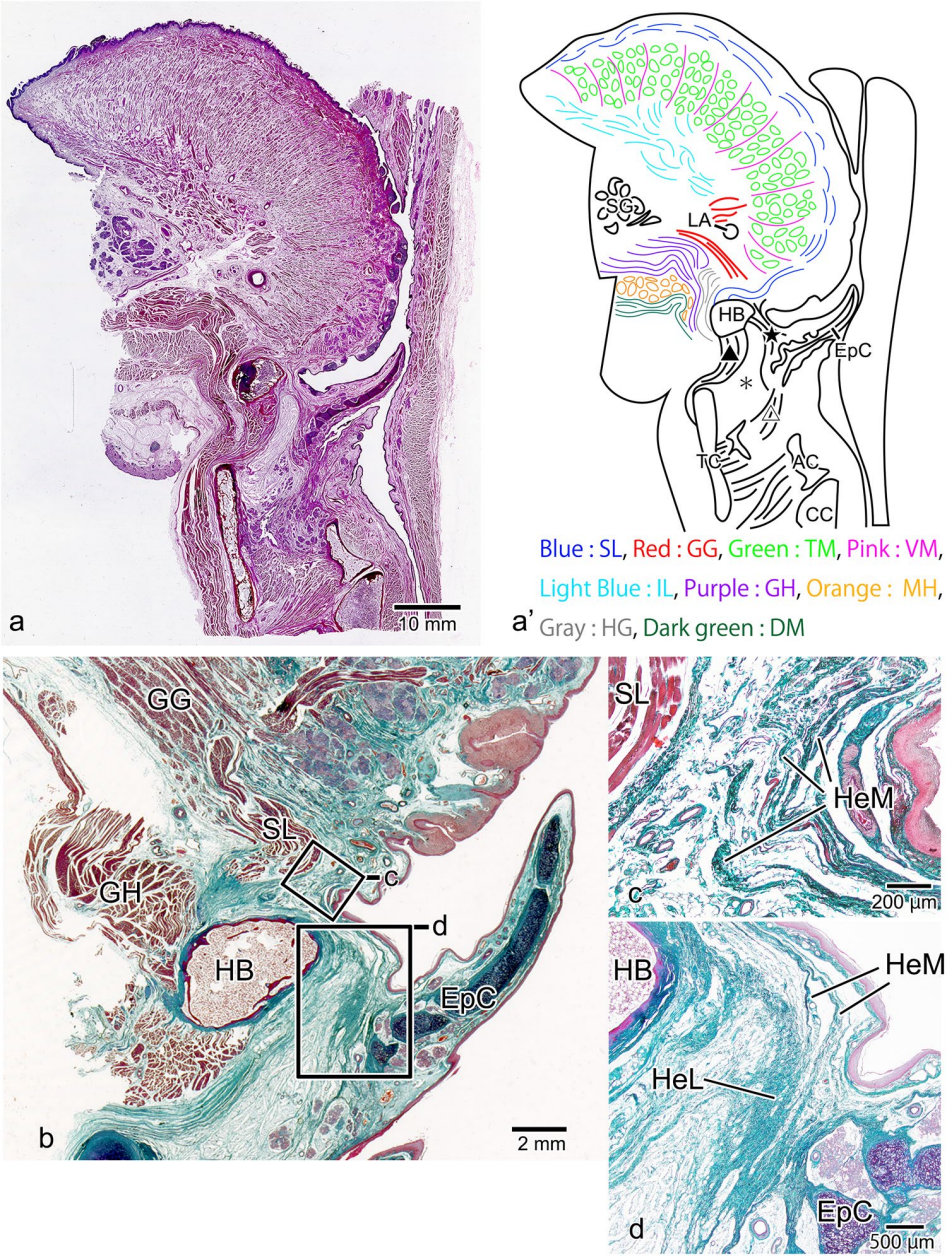
Sagittal Sect. 6-mm lateral to the midline. In the surface layer of the tongue, the course of the superior longitudinal (SL: Blue) lingual muscle is similar to that in Fig. 3. Within the tongue, however, the genioglossus (GG: Red) muscle is limited to the posterior-most part, and the majority of the tongue is formed by the vertical muscle (VM: Pink) and transverse muscle (TM: Green) of the tongue and the inferior longitudinal (IL: Light Blue) lingual muscle (**a**, a’). In the magnified image of the tongue root, the SL lingual muscle exhibits greater continuity than in Fig. 3, and it completely separates the GG muscle from the epiglottic cartilage (EpC) (**b**). Because of this, the hyoepiglottic membrane (HeM) converges before it reaches the SL lingual muscle (**c**). The HeM merges with the hyoepiglottic ligament (HeL) and attaches to the epiglottic cartilage (**d**). LA: lingual artery, SG: sublingual gland, TC: thyroid cartilage, *CC* cricoid cartilage, *AC* arytenoid cartilage, *GH* geniohyoid muscle (Purple), *M*: mylohyoid muscle (Orange), *HG* hyoglossus muscle (Gray), *DM* digastric muscle (Dark green), ★: hyoepiglottic ligament, filled triangle: thyrohyoid ligament; open triangle: thyroepiglottic ligament; *: pre-epiglottic adipose tissue

In a sagittal Sect. 6-mm lateral to the median sagittal section, the superior longitudinal lingual muscle was observed in a similar area to that of Fig. 4. The genioglossus muscle was only evident in the posterior-most part, with the majority of the tongue formed by the vertical muscle and transverse muscle of the tongue and the inferior longitudinal lingual muscle (Fig. 5a, a’). In the magnified image of the tongue root, there was an epithelial layer on the superior-dorsal side of the hyoid bone, with intervening very thin submucosal tissue. Compared with Fig. 4, the superior longitudinal lingual muscle extending from the superior edge of the hyoid bone had become continuous. This continuous superior longitudinal lingual muscle completely blocked the ingress of the genioglossus muscle into the larynx (Fig. 5b). Because of this, the hyoepiglottic membrane had converged with the epiglottal-side fascia of the superior longitudinal lingual muscle, and no junction with the genioglossus muscle was observed (Fig. 5c). From the dorsal side of the hyoid bone, the hyoepiglottic ligament formed a thick-fiber bundle, which merged with the hyoepiglottic membrane partway through and ran toward the base of the epiglottic cartilage (Fig. 5d).

## Histological Analysis of the Tongue and Epiglottis Observed in Horizontal and Coronal Sections

### Horizontal Section

**Fig. 6:**
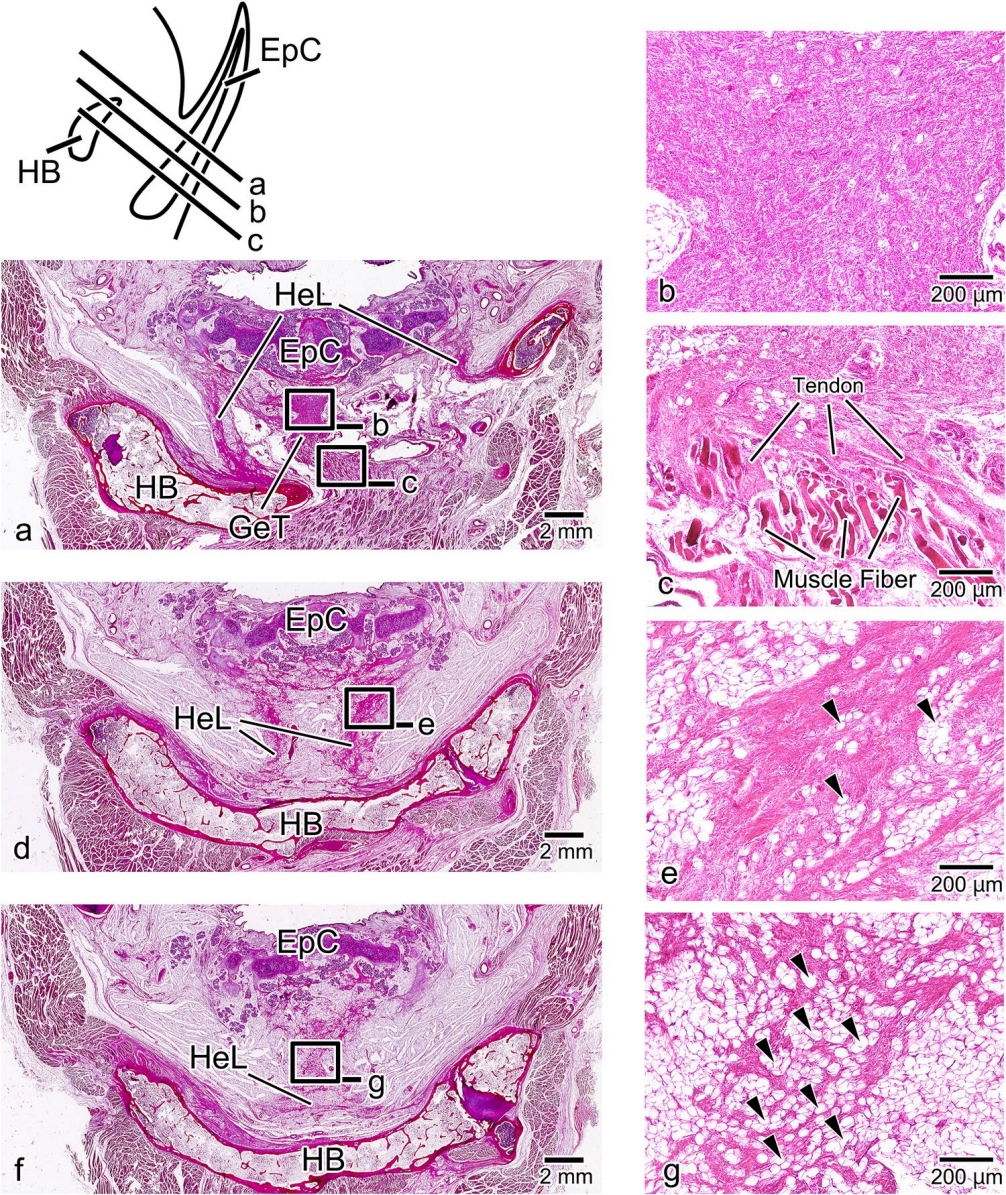
Histological observations of the tongue and epiglottis in horizontal section. In the upper horizontal section, the glossoepiglottic tendon (GeT) is evident at the midline, with a pair of hyoepiglottic ligaments (HeLs) lateral to it on either side (**a**). The GeT is the thickest, highly dense fiber bundle and contains almost no adipose tissue (**b**). The GeT forms a muscle–tendon junction with the muscle fibers of the tongue root (**c**). In the intermediate horizontal section, the GeT has disappeared, and the bilateral HeLs have moved toward the midline (**d**). The HeL at this level is a fiber bundle that contains internal adipose tissue (**e**, arrowhead). In the lower horizontal section, the bilateral HeLs have merged at the midline, forming a single fiber bundle (**f**). The HeL at this level contains a large amount of internal adipose tissue and is the sparsest fiber bundle (**g**, arrowhead). HB: hyoid bone, EpC: epiglottic cartilage

In the upper horizontal section, the median glossoepiglottic tendon passed through the upper part of the body of the hyoid bone to reach the tongue root, with the pair of hyoepiglottic ligaments at each side running between the greater cornua of the hyoid bone and the epiglottic cartilage (Fig. 6a). The glossoepiglottic tendon was the thickest, highly dense fiber bundle and contained almost no adipose tissue (Fig. 6b). This tendon formed a muscle–tendon junction with muscle fibers considered to be the genioglossus muscle of the tongue root (Fig. 6c). In the intermediate horizontal section, the glossoepiglottic tendon had disappeared, and the bilateral hyoepiglottic ligaments had moved toward the midline (Fig. 6d). In this cross-section, the hyoepiglottic ligament was a fiber bundle containing internal adipose tissue (Fig. 6e). In the lower horizontal section, the bilateral hyoepiglottic ligaments had merged to become a single median fiber bundle (Fig. 6f). In this cross-section, the hyoepiglottic ligament was the thin-fiber bundle, and it contained a large amount of internal adipose tissue (Fig. 6g).

### Coronal Section

**Fig. 7:**
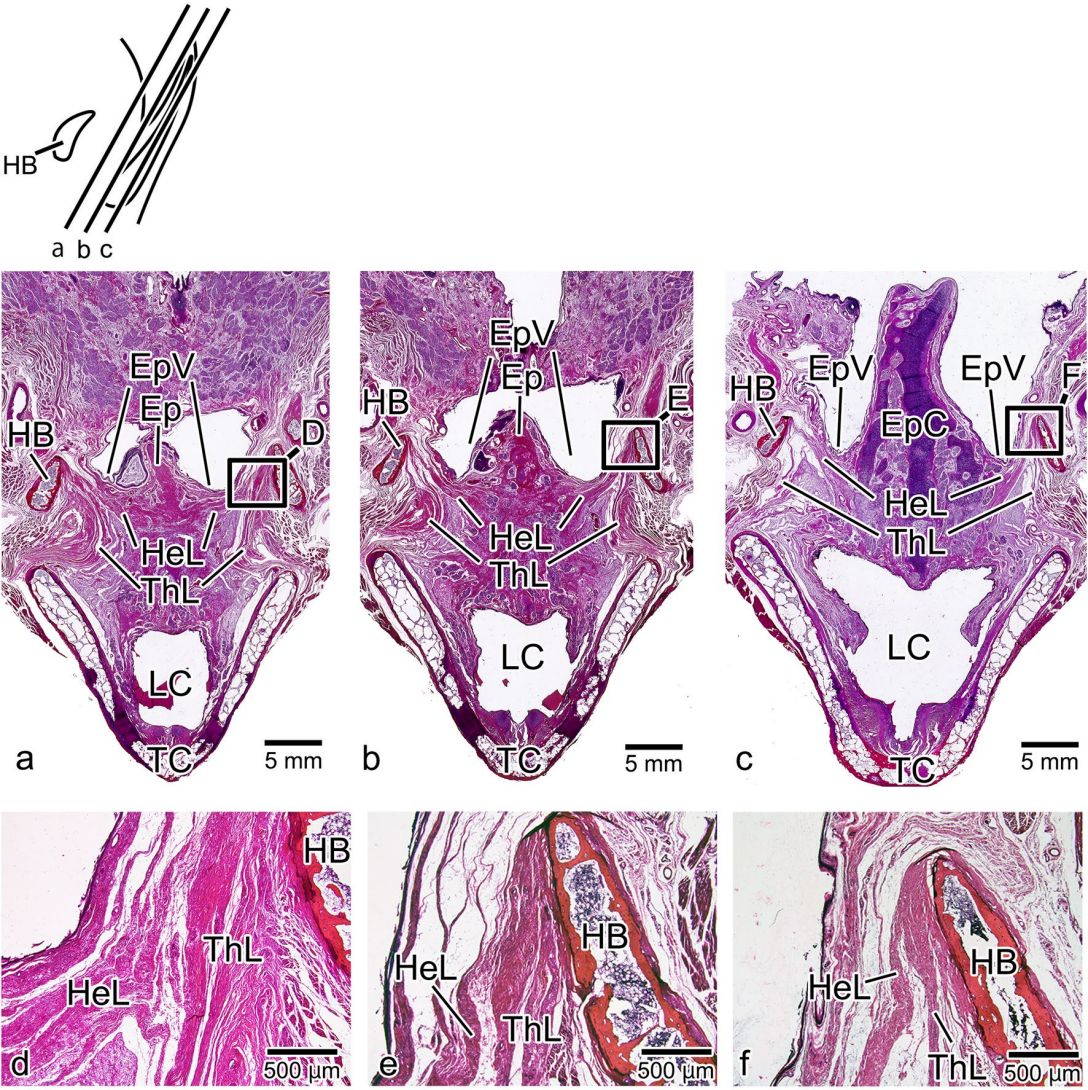
Histological observations of the tongue and epiglottis in coronal section. The epiglottis (EP) is evident at the midline, with the epiglottic valleculae (EpVs) on both sides, the hyoid bone (HB) on the lateral side, and the thyroid cartilage (TC) below. The thyrohyoid ligament (ThL) runs between the HB and the TC, and the hyoepiglottic ligament (HeL) runs between the HB and the EP (**a**, **b**, **c**). In the anterior part, the ThL attaches to the HB, and the HeL to the ThL (**d**, **e**). Further posterior, however, the ThL changes to a fine fiber bundle, and the HeL extends to the superior edge of the HB (**f**). EpC: epiglottic cartilage, LC: laryngeal cavity

In coronal section, the epiglottis was evident at the midline, with the valleculae on both sides, the hyoid bone on the lateral side, and the thyroid cartilage below. From the hyoid bone, the thyrohyoid ligament ran toward the thyroid cartilage, and the hyoepiglottic ligament ran toward the epiglottis in the inferior medial direction. These structures did not undergo any major changes from the anterior to the posterior, with the hyoepiglottic ligament always forming the submucosal tissue of the epiglottic valleculae (Fig. 7a–c). Anterior to the epiglottic cartilage, the thyrohyoid ligament was broadly attached to the hyoid bone, and the hyoepiglottic ligament contacted the thyrohyoid ligament (Fig. 7d, e). However, in the cross-section containing the epiglottic cartilage, the thyrohyoid ligament had changed to a fine fiber bundle, and the hyoepiglottic ligament was extended to the upper edge of the hyoid bone (Fig. 7f).

## Morphology of the Glossoepiglottic Tendon Insertion into the Epiglottic Cartilage

**Fig. 8:**
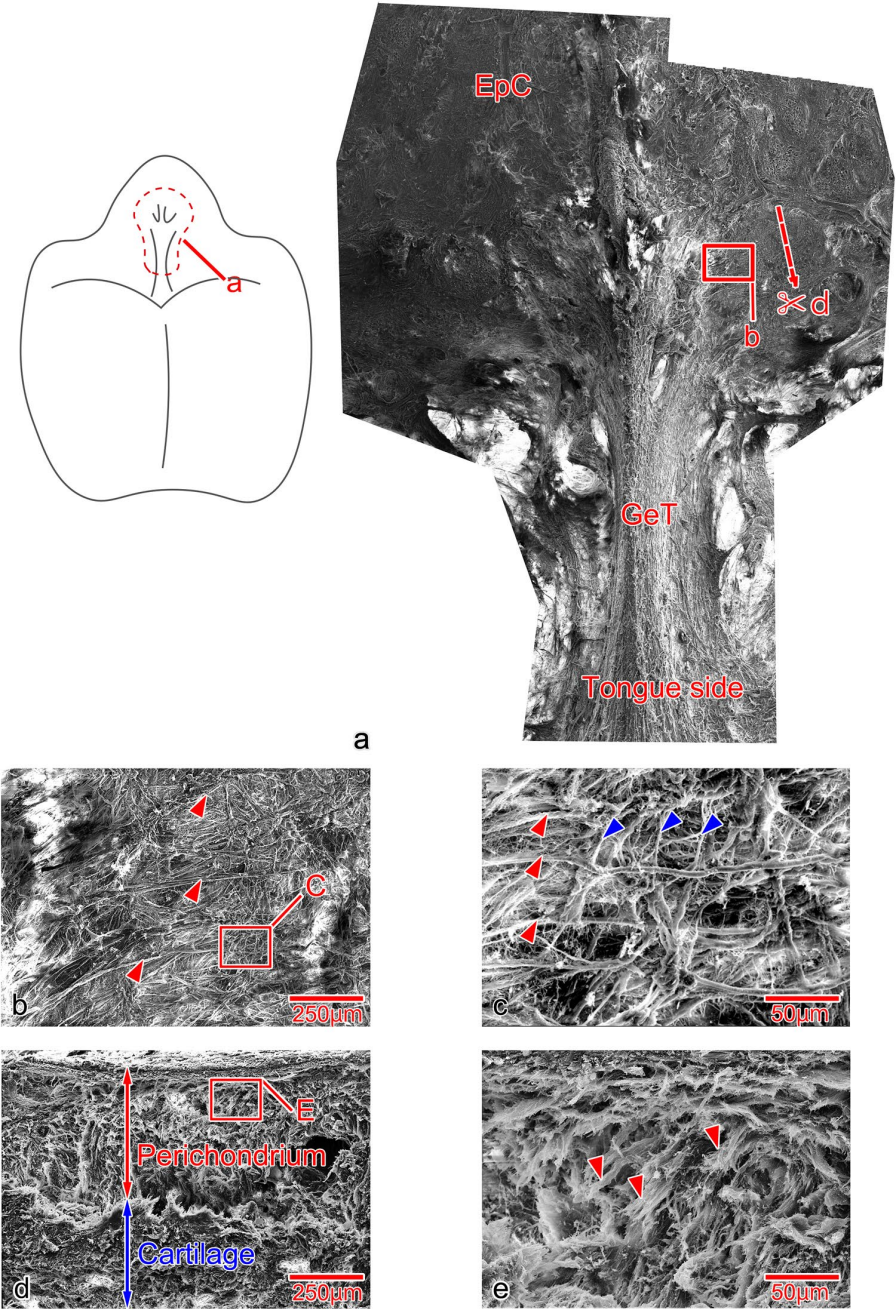
Morphology of the insertion of the GeT into the EpC. **a** The tongue and epiglottis viewed from above. The glossoepiglottic tendon (GeT) is composed of numerous collagen fiber bundles that radiate out from the center of the epiglottic cartilage (EpC). **b** The collagen fibers of the GeT run along the surface of the EpC (red arrowhead). **c** The terminal of the GeT (red arrow) and the fibers of the perichondrium of the EpC (blue arrowhead) form bridge structures in a reticular pattern. **d** Cross-sectional view of the insertion. Perichondrium and cartilage matrix are present from the superficial layer. **e** The collagen fibers of the GeT are limited to the perichondrium (red arrowhead)

When the tongue and epiglottis were viewed from above, the glossoepiglottic tendon was composed of numerous collagen fiber bundles that radiated out from the center of the epiglottic cartilage (Fig. 8a). These collagen fibers ran along the surface of the epiglottic cartilage (Fig. 8b, red arrowhead), and at their terminals, they formed bridges in a reticular pattern with the fine fibers of the perichondrium of the epiglottic cartilage (Fig. 8c, red and blue arrowheads). In cross-sectional view, perichondrium was present in the superficial layer of the insertion site and cartilage matrix in its deep layer (Fig. 8d), and the collagen fibers of the glossoepiglottic tendon were only evident in the perichondrium (Fig. 8e, red arrowhead).

## Age-Related and Sex-Related Differences

The findings seen in this investigation were observed in all 19 cadavers. However, because of differences in the size of the epiglottis between cadavers, the same structures were not always observed at specific distances apart. The epiglottis tended to be larger in men and smaller in women. The slit in the superior part of the hyoid bone tended to be shorter when the position of the larynx was higher and longer when it was lower. This may be related to age-associated ptosis of the larynx, but this was not clear from the results of the present study.

## Discussion

**Fig. 9:**
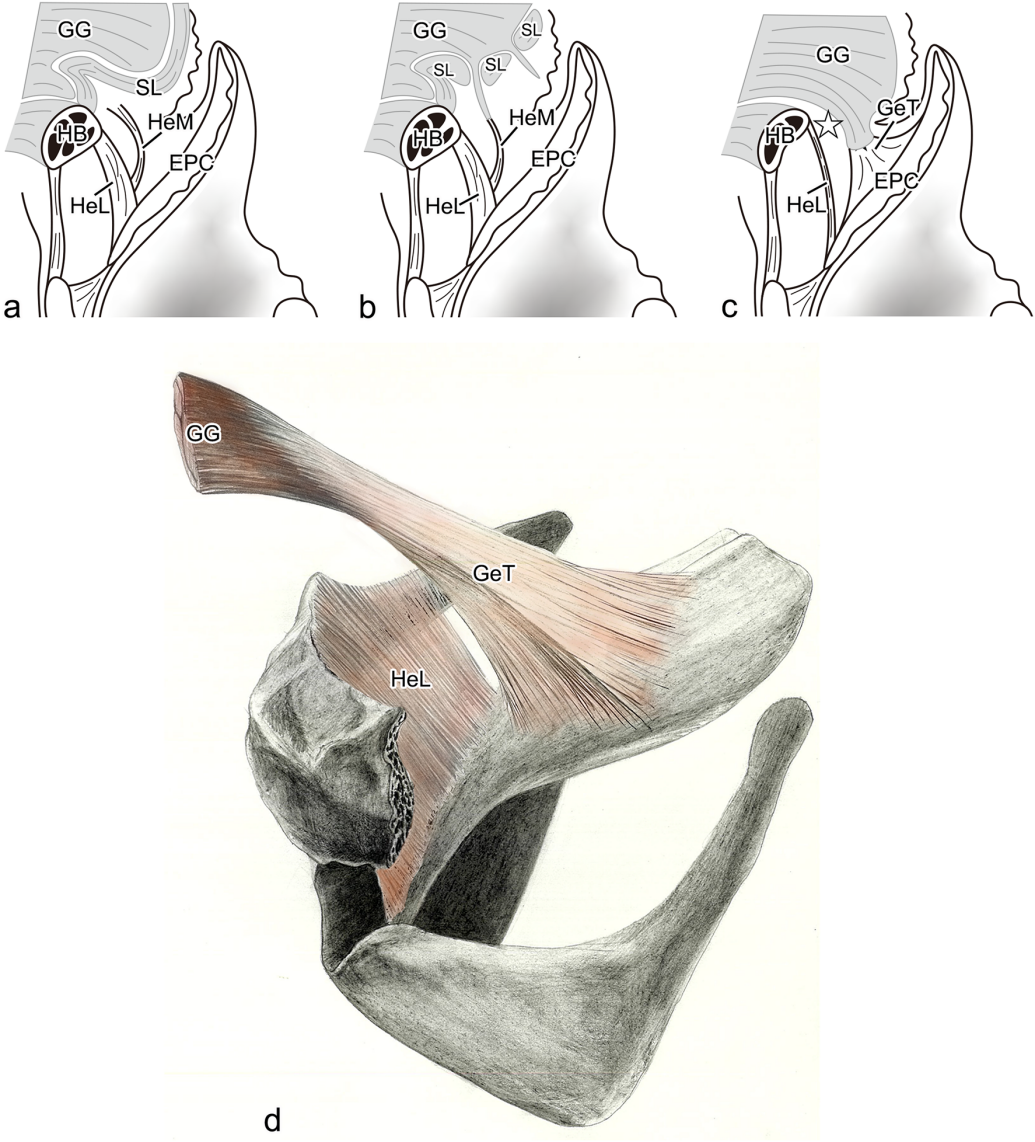
Diagrams of the three-dimensional courses of the HeL, HeM, and GeT. **a** Diagram of a lateral sagittal section of the epiglottis. The superior longitudinal (SL) lingual muscle runs continuously from the hyoid bone (HB), blocking any contact between the genioglossus (GG) muscle and the hyoepiglottic membrane (HeM). **b** Diagram of a mid-lateral sagittal section of the epiglottis. The HeM, which has branched off from the top of the hyoepiglottic ligament (HeL), forms a muscle–tendon junction with the GG muscle, which penetrates between the fragmented SL lingual muscles. **c** Diagram of a median sagittal section of the epiglottis. It has a two-layer structure divided by a slit (☆), with the upper layer consisting of the glossoepiglottic tendon (GeT) that joins the GG muscle and the lower layer formed by the fine HeL. **d** Three-dimensional diagram of the upper fibers in the pre-epiglottic space

In the long history of anatomy to date, very few studies have discussed the relationship between the lingual muscles and the epiglottis in morphological terms [17, 18]. The structure of the hyoepiglottic ligament, which divides the tongue from the epiglottis, has therefore yet to be completely explained. Van Daele showed that the hyoepiglottic ligament starts from the lateral side of the lingual surface of the epiglottis and is attached to the greater cornu of the hyoid bone and the fascia of the lingual muscles of its upper portion. We observed similar results in the epiglottis in lateral sagittal section, and additionally observed the novel finding that the fascia is that of the superior longitudinal lingual muscle (Fig. 9a). The fact that the superior longitudinal lingual muscle attaches to the hyoid bone is also a new discovery, and this suggested that a simpler function of the hyoepiglottic ligament is exerted because the superior longitudinal lingual muscle prevents the genioglossus muscle from progressing backward. Reidenbach defined the hyoepiglottic ligament as a two-layer ligament and showed that the lower layer is attached to the periosteum of the superior edge of the hyoid bone, whereas the upper layer spreads out within the muscle tissue of the tongue root. Since the upper layer is also a semi-independent fiber bundle that is also found in the free part of the epiglottic cartilage, he named this the hyoepiglottic membrane. We also made similar observations in the mid-lateral section of the epiglottis and also found that the genioglossus muscle, which runs posteriorly while breaking through the superior longitudinal lingual muscle bundles, forms a muscle–tendon junction with the hyoepiglottic membrane (Fig. 9b). The major difference between Van Daele’s and Reidenbach’s results and ours is the structure of the median epiglottis. In the median section of the epiglottis, a tendon that forms a thick-muscle–tendon junction with the genioglossus muscle is broadly attached to the lingual side of the epiglottic cartilage. Because it formed a narrow slit with the thin-hyoepiglottic ligament directed toward the base of the epiglottic cartilage, we recognized this tendon as a completely independent fiber and have therefore named it the glossoepiglottic tendon (Fig. 9c). From the above results, the glossoepiglottic tendon forms a thick-muscle–tendon junction with the genioglossus muscle in the median epiglottis and is broadly attached to the middle of the lingual surface of the epiglottic cartilage. The hyoepiglottic ligament, on the other hand, runs between the hyoid bone and the epiglottis, and it was found to attach in a V shape directed from the lateral margin of the epiglottis to its median base (Fig. 9d). The glossoepiglottic tendon that was discovered has never been reported in anatomical studies of the larynx, such as those by Van Daele and Reidenbach. This new discovery required three unique research methods. The first was that the observation target was from the epiglottis to the entire tongue. This made it possible to more clearly observe the relationship between the epiglottic cartilage and the lingual muscle at the base of the tongue. The second was that sagittal, horizontal, and forehead cuts were examined; three cross-sections clarified three-dimensional fiber bundle orientation. The third was that continuous sections were prepared at equal intervals. For the larynx, where the structure changes significantly in a small area, continuous sections were effective.

The basic components required of a motor organ in the living body are the muscles that provide its motive power, the tendons that transmit this force, and the skeleton to exert its action. Among these components, the connections between tendons and bones are known as entheses [21]. Entheses are histologically classified into three different types: periosteal insertions, fibrous entheses, and fibrocartilage entheses. In periosteal insertions, the collagen fibers of the tendon are inserted into the periosteum; in fibrous entheses, they pass through the periosteum and are inserted into the bone matrix; and in fibrocartilage entheses, they pass through the layer of fibrocartilage on the surface of the bone and are inserted into the bone matrix [22]. In the masticatory muscles of the head and neck in particular, these three types of enthesis are all found together in a single muscle insertion [23, 24]. In the present experiments, however, the morphology of the insertion of the glossoepiglottic tendon into the epiglottic cartilage resembled that of a periosteal insertion, and no fibrous or fibrocartilage enthesis was observed. In addition, the skeleton that is the point of action is not hard bony tissue, but is composed of elastic cartilage, the softest form of cartilage, making this insertion unlike the previously reported concepts of entheses. These results suggested that in cartilage that undergoes significant distortion and periosteal insertions, which are attached across a broad area as if sewn into the perichondrium, may be better at preventing deformation of the cartilage when it functions are fibrous or fibrocartilage entheses, which are fitted deeply into a small spot.

Experiments concerning the computational analysis of swallowing mechanics use videofluoroscopy or barium X-rays in modified barium swallow studies to enable the amount of movement of anatomical structures to be measured and subjected to vector analysis or quantitative evaluation [25–27]. On the basis of previous computational analysis of swallowing mechanics, the first epiglottic movement was formerly thought to occur in tandem with the movements of the hyoid bone and larynx [14–16]. Recently, however, numerous studies have reported that the main component of the first epiglottic movement is in fact the backward movement of the tongue root [28–30]. Orsbon et al*.* further used electromyography to show that the genioglossus muscle relaxes and the geniohyoid muscle contracts during the first epiglottic movement [31]. In the present study, the slit that was identified in median sagittal section was shown to be a structure that can separate the movements of the genioglossus muscle and the geniohyoid muscle. That is, the glossoepiglottic tendon in the upper layer of the slit transmits the relaxation of the genioglossus muscle to the upper part of the epiglottis, and the hyoepiglottic ligament in the lower layer of the slit transmits the contraction of the geniohyoid muscle to the lower part of the epiglottis. This suggests that these two opposing movements may generate a rotational force that causes the retroversion of the epiglottis. We also considered that the relaxation of the genioglossus muscle and the glossoepiglottic tendon may be involved in the previously reported extension of the hyoepiglottic ligament during the first epiglottic movement. In the second epiglottic movement, it is the forward movement of the hyoid bone that reportedly causes the hyoepiglottic ligament on the lateral side to draw the tip of the epiglottis forward and downward, so that it completely covers the laryngeal aperture [17]. In this study, the hyoepiglottic ligament was also identified as a broad, strong ligament in the lateral sagittal section, a result that supports this mechanism for the second epiglottic movement. However, the effects on the second epiglottic movement of pressure on the posterior wall of the pharynx and of the thyroepiglottic and aryepiglottic muscles must also be taken into account [15].

At rest, the swallowing reflex is inhibited by the filling of the valleculae with saliva close to the tongue root [32]. This function reportedly prevents the swallowing reflex from becoming excessive during sleep at night, maintaining the pharynx and larynx in a relaxed state, but the detailed mechanism of this movement has yet to be identified [33]. In this study, the glossoepiglottic tendon was found to be located in the submucosa of the glossoepiglottic fold, and the hyoepiglottic ligament was found to be located in the submucosa of the epiglottic valleculae. From these results, at rest, the glossoepiglottic tendon is tensed and the glossoepiglottic fold is clearly evident, enabling saliva to move smoothly into the epiglottic valleculae. This suggests that, in its resting position, the hyoid bone and the hyoepiglottic ligament form a deep epiglottic valleculae that collects saliva.

The genioglossus muscle is classified as an extrinsic lingual muscle, and it is found as a pair of muscles on either side of the lingual septum. The genioglossus muscle runs in the sagittal direction, tracing an arc from the mental spine toward the tip of the tongue, the dorsum of the tongue, and the tongue root [34, 35]. In this course, the posterior portion running toward the root of the tongue is thicker than the rest of the muscle, and it was previously regarded as a different muscle separated by connective tissue [36]. The posterior part of the genioglossus muscle also functions to move the hyoid bone and tongue root forward, dilating the upper airway, and numerous studies have described its involvement in respiration and swallowing actions [37–40]. In addition, many studies of muscle attachment have been reported [41, 42], along with a paper describing a short-term experiment on the tongue muscle in mice [43]. In terms of the human oral cavity, there are reports on the soft palate and pharynx [44, 45]. However, no study has yet described the morphology of the terminus of the posterior part of the genioglossus muscle, and the mechanism of upper airway dilation remains unknown. In the present study, the existence of the glossoepiglottic tendon was identified, and the terminus of the genioglossus muscle was found not to be the hyoid bone. From these findings, we consider that the contraction of the posterior part of the genioglossus muscle directly draws the epiglottis forward, and this dilates the upper airway. These results suggest that this function works at rest and during sleep in particular and that the genioglossus muscle plays a role as an anti-gravity muscle to keep the upper airway consistently open.

## Conclusion

Gross and microscopic observations of the submucosal structures from the tongue to the larynx of 20 elderly cadavers were performed. As a result, in the upper layer of the hyoepiglottic ligament, a new tendon that connects the genioglossus muscle and the epiglottic cartilage, named the glossoepiglottic tendon, was discovered. Interestingly, there was a slit between the hyoepiglottic ligament and the glossoepiglottic tendon that can separate the movements of the two fiber bundles. These results suggest that the genioglossus muscle may be involved in keeping the epiglottis upright and in its retroversion. Therefore, the function of the genioglossus muscle is a very important item to evaluate for rehabilitation of dysphagia.
